# Supplementary figures and images for: Elevated temperature inhibits SARS-CoV-2 replication in respiratory epithelium independently of IFN-mediated innate immune defenses
Source: PLoS Biol. 2021 Dec 21;19(12):e3001065. doi: 10.1371/journal.pbio.3001065 (PMC8765667; doi:10.1371/journal.pbio.3001065)

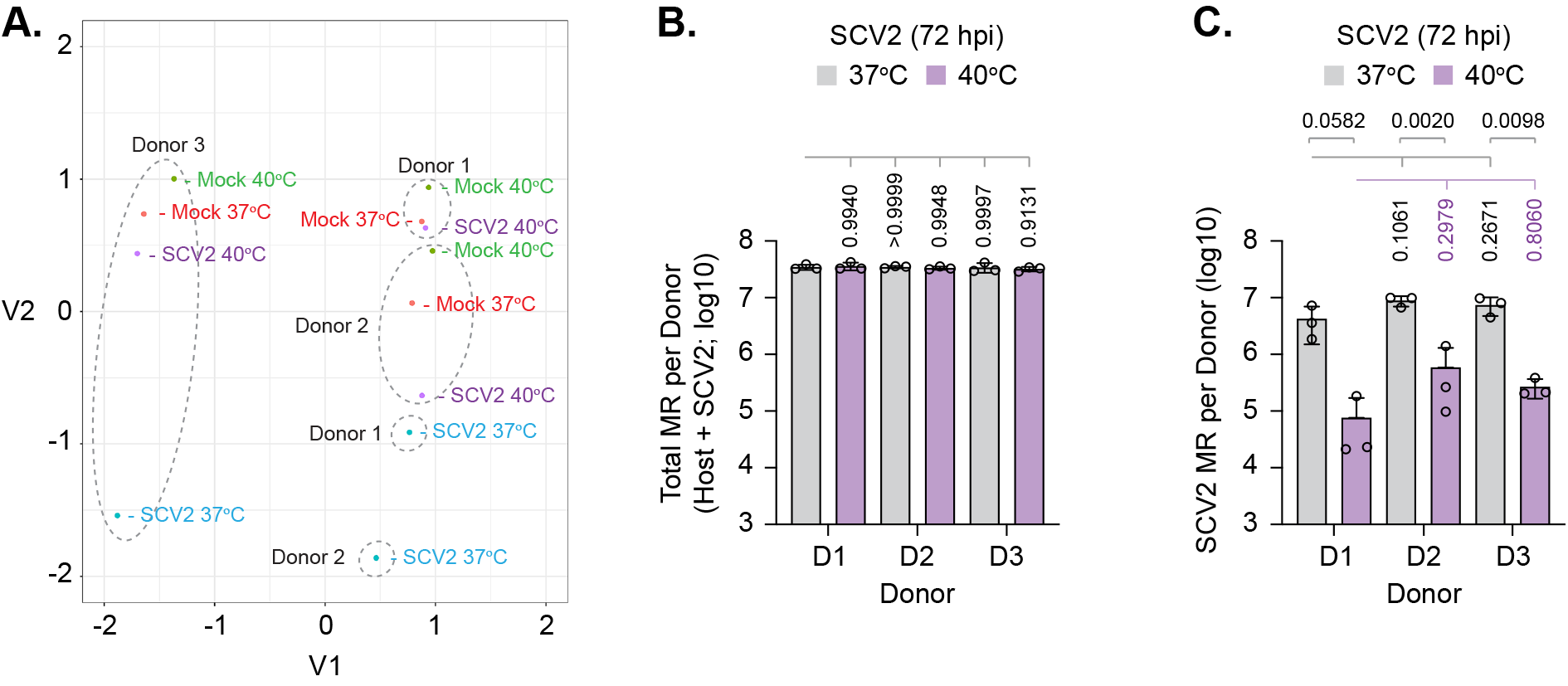

Supplement: S1 Fig — Ciliated respiratory cultures differentiated from primary HBEp cells isolated from 3 independent donors (donor 1, male Caucasian aged 63 years; donor 2, Hispanic male aged 62 years; donor 3, Caucasian female aged 16 years; all nonsmokers) were incubated at 37 or 40°C for 24 h prior to mock (media only) or SARS-CoV-2 (SCV2; MOI 0.05, 104 PFU/Tissue) infection. Tissues were incubated at their respective temperatures for 72 h prior to RNA extraction and RNA-Seq. (A) PCA analysis of mean CPM values derived from host transcriptome mapping of donor samples (dotted circles; donors 1 to 3) per experimental condition (as indicated). (B) Total MR (host + SARS-CoV-2) counts of donor (D1 to D3) infected samples at 37 or 40°C (as indicated); p-values shown, one-way ANOVA. (C) SARS-CoV-2 MR counts of donor (D1 to D3) infected samples at 37 or 40°C; p-values shown; top, unpaired two-tailed t test; bottom, one-way ANOVA. (A to C) RNA-Seq data derived from RNA isolated from 3 independent biological experiments per donor. Raw values presented in S9 Data. CPM, counts per million; HBEp, human bronchiolar epithelial; MR, mapped read; PCA, principle component analysis; PFU, plaque-forming unit; RNA-Seq, RNA sequencing; SARS-CoV-2, Severe Acute Respiratory Syndrome Coronavirus 2. (TIF) [file pbio.3001065.s001.tif]

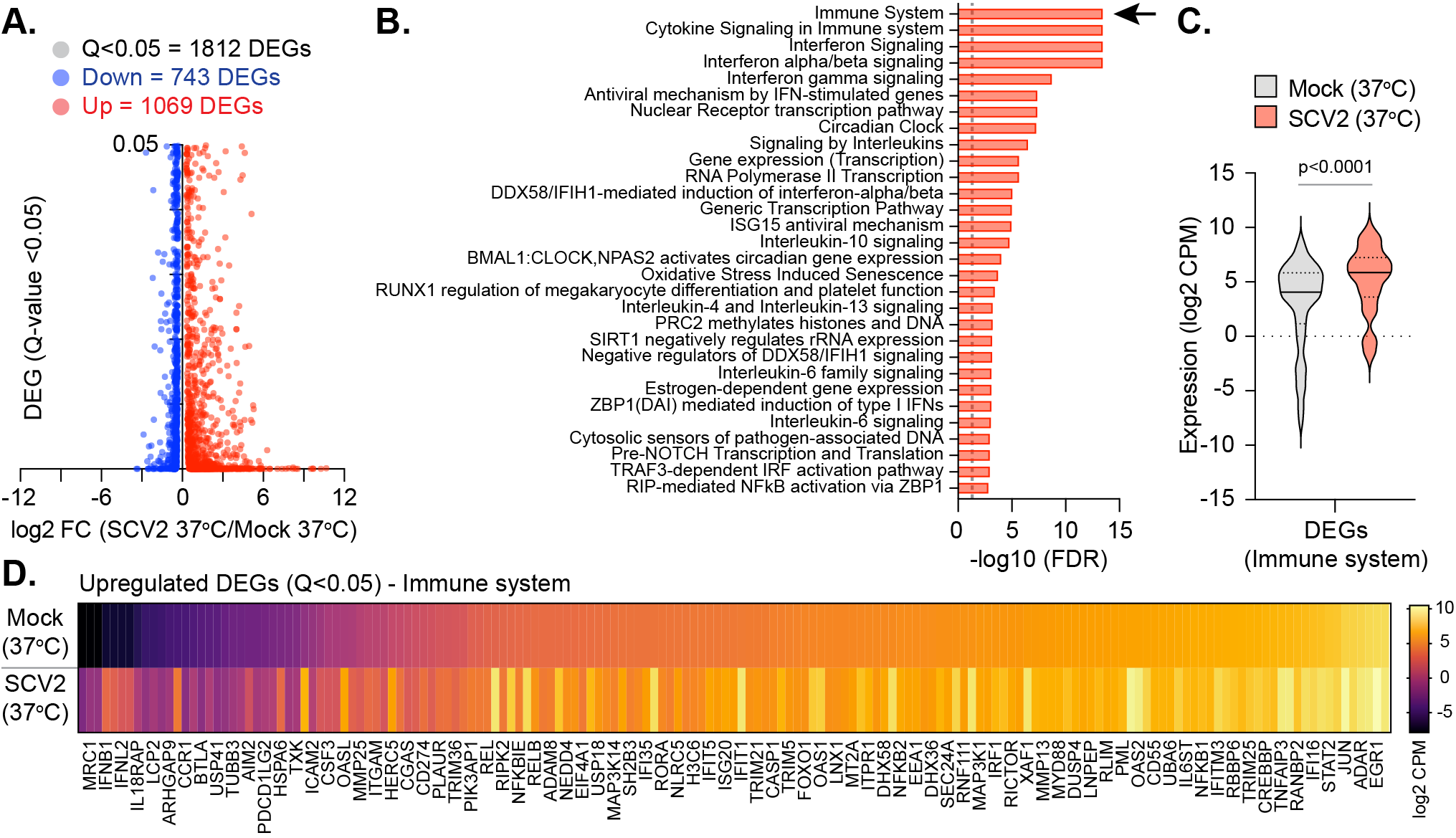

Supplement: S2 Fig — Ciliated respiratory cultures differentiated from primary HBEp cells isolated from 3 independent donors (donor 1, male Caucasian aged 63 years; donor 2, Hispanic male aged 62 years; donor 3, Caucasian female aged 16 years; all nonsmokers) were mock (media only) or SARS-CoV-2 (SCV2; MOI 0.05, 104 PFU/Tissue) infected at 37°C for 72 h prior to RNA extraction and RNA-Seq. (A) Scatter plots showing high-confidence (Q < 0.05) DEG transcripts identified between mock and SARS-CoV-2–infected cultures; up-regulated DEGs, red circles; down-regulated DEGs, blue circles. (B) Reactome pathway analysis of mapped up-regulated DEGs. Top 30 up-regulated (FDR < 0.05) pathways shown (red bars; plotted as −log10 FDR). Dotted line, threshold of significance (−log10 FDR of 0.05). (C) Expression profile (log2 CPM) of immune system–related DEGs (R-HSA168256; arrow in B). Black line, median; dotted lines; fifth and 95th percentile range; p-value shown, paired two-tailed t test. (D) Expression levels (log2 CPM) of individual immune system DEGs (arrow in B). Every second gene labeled. (A to D) RNA-Seq data derived from RNA isolated from 3 donors (donors 1 to 3) per sample condition from 3 independent biological experiments per donor. Raw values presented in S3 and S9 Data. CPM, counts per million; DEG, differentially expressed gene; FDR, false discovery rate; HBEp, human bronchiolar epithelial; IFN, interferon; PFU, plaque-forming unit; RNA-Seq, RNA sequencing; SARS-CoV-2, Severe Acute Respiratory Syndrome Coronavirus 2. (TIF) [file pbio.3001065.s002.tif]

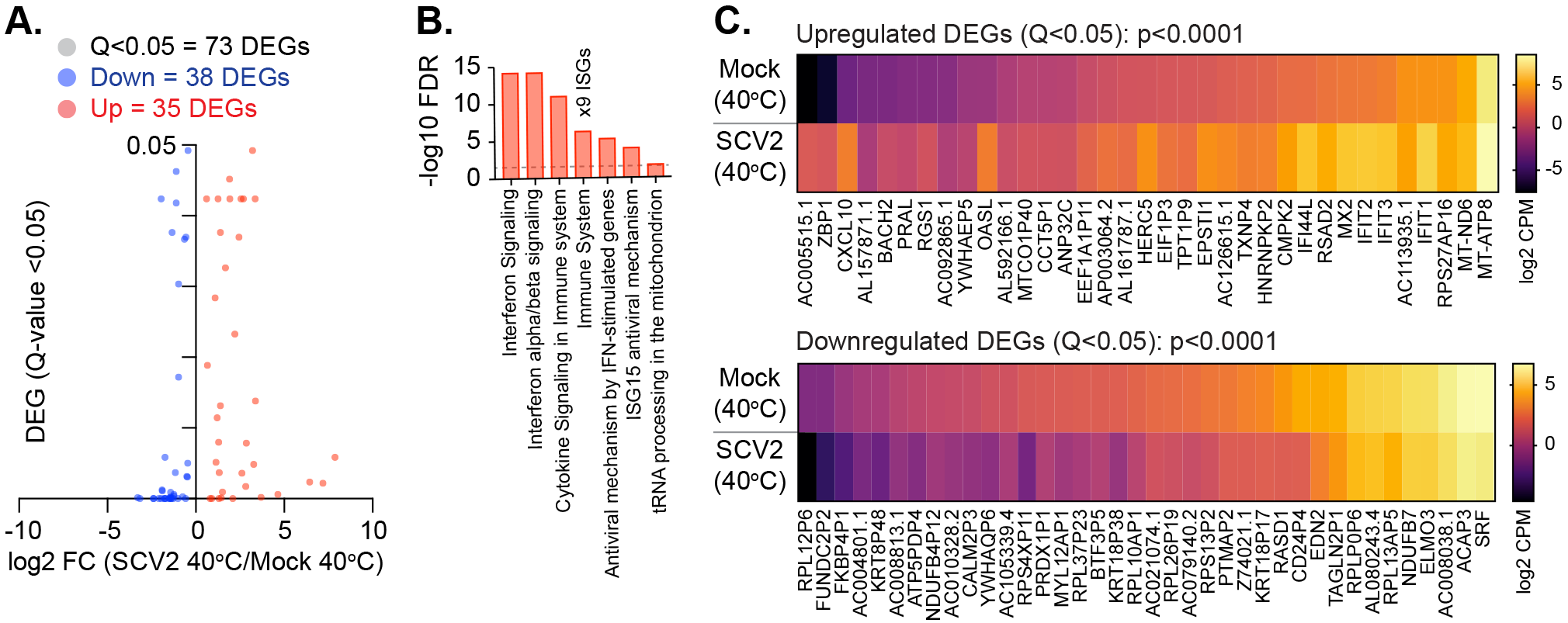

Supplement: S3 Fig — Ciliated respiratory cultures differentiated from primary HBEp cells isolated from 3 independent donors (donor 1, male Caucasian aged 63 years; donor 2, Hispanic male aged 62 years; donor 3, Caucasian female aged 16 years; all nonsmokers) were incubated at 40°C for 24 h prior to mock (media only) or SARS-CoV-2 (SCV2; MOI 0.05, 104 PFU/Tissue) infection and continued incubation at 40°C. Tissues were harvested at 72 h for RNA extraction and RNA-Seq. (A) Scatter plots showing high-confidence (Q < 0.05) DEG transcripts identified between mock and SARS-CoV-2–infected respiratory cultures at 40°C; up-regulated DEGs, red circles; down-regulated DEGs, blue circles. (B) Reactome pathway analysis of mapped up-regulated DEGs. Up-regulated pathways shown (red bars; plotted as −log10 FDR). Dotted line, threshold of significance (−log10 FDR of 0.05). (C) Expression values (log2 CPM) of Reactome mapped DEGs (identified in A); p-values shown, paired two-tailed t test. (A to C) RNA-Seq data derived from RNA isolated from 3 donors (donors 1 to 3) per sample condition from 3 independent biological experiments per donor. Raw values presented in S4 and S9 Data. CPM, counts per million; DEG, differentially expressed gene; FDR, false discovery rate; HBEp, human bronchiolar epithelial; IFN, interferon; ISG, IFN-stimulated gene; PFU, plaque-forming unit; RNA-Seq, RNA sequencing; SARS-CoV-2, Severe Acute Respiratory Syndrome Coronavirus 2. (TIF) [file pbio.3001065.s003.tif]

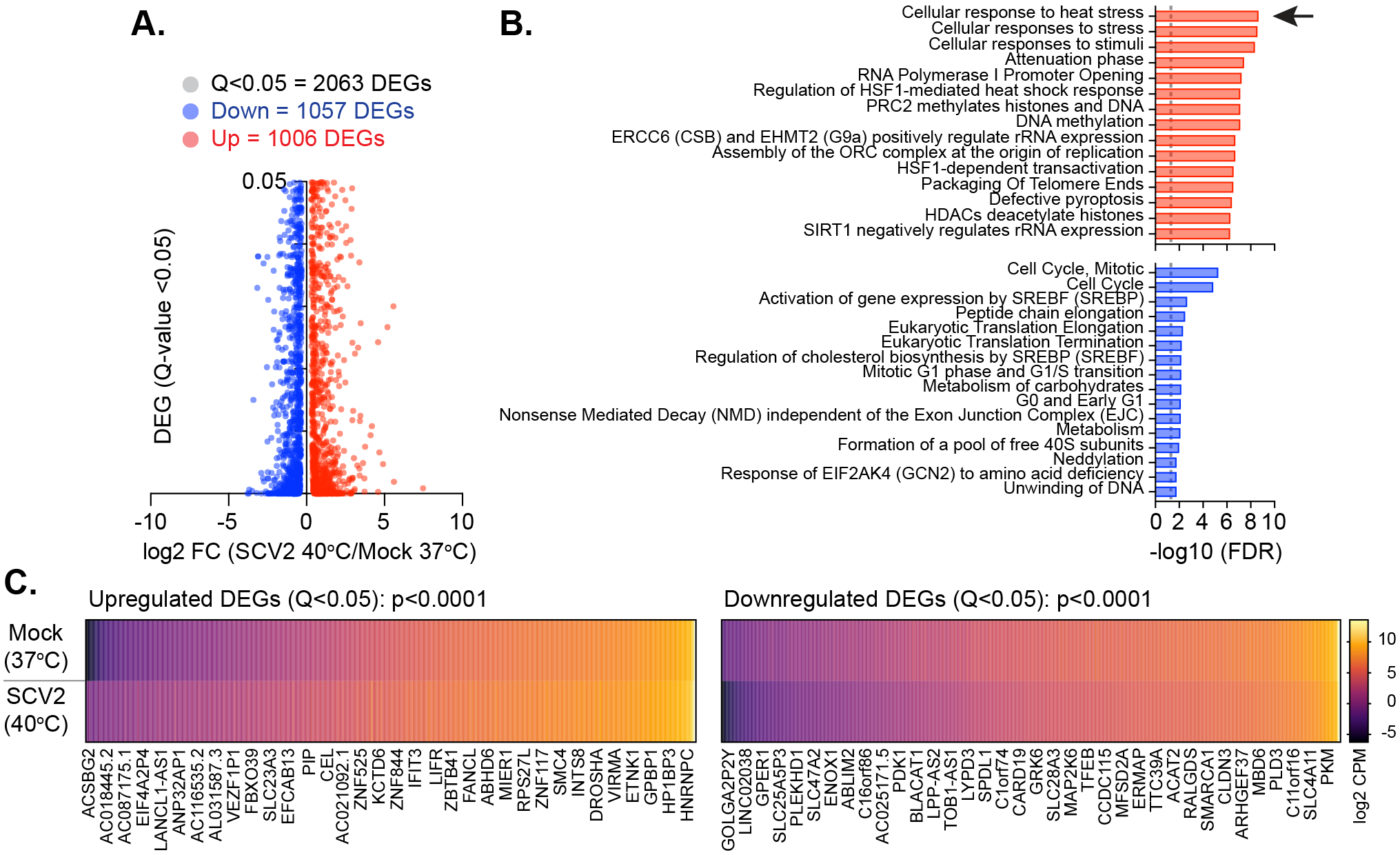

Supplement: S4 Fig — Ciliated respiratory cultures differentiated from primary HBEp cells isolated from 3 independent donors (donor 1, male Caucasian aged 63 years; donor 2, Hispanic male aged 62 years; donor 3, Caucasian female aged 16 years; all nonsmokers) were incubated at 37 or 40°C for 24 h prior to mock (media only) or SARS-CoV-2 (SCV2; MOI 0.05, 104 PFU/Tissue) infection and continued incubation at their respective temperatures (as indicated). Tissues were harvested at 72 h for RNA extraction and RNA-Seq. (A) Scatter plots showing high-confidence (Q < 0.05) DEG transcripts identified between mock (37°C) and SARS-CoV-2–infected (40°C) respiratory cultures; up-regulated DEGs, red circles; down-regulated DEGs, blue circles. (B) Reactome pathway analysis of mapped DEGs. Top 15 up-regulated and down-regulated (FDR < 0.05; red and blue bars, respectively; plotted as −log10 FDR) pathways shown. Dotted line, threshold of significance (−log10 FDR of 0.05). Black arrow, identification of cellular response to heat stress pathway. (C) Expression values (log2 CPM) of Reactome mapped DEGs; p-values shown, paired two-tailed t test. Every 30th gene labeled. (A to C) RNA-Seq data derived from RNA isolated from 3 donors (donors 1 to 3) per sample condition from 3 independent biological experiments per donor. Raw values presented in S5 and S9 Data. CPM, counts per million; DEG, differentially expressed gene; FDR, false discovery rate; HBEp, human bronchiolar epithelial; PFU, plaque-forming unit; RNA-Seq, RNA sequencing; SARS-CoV-2, Severe Acute Respiratory Syndrome Coronavirus 2. (TIF) [file pbio.3001065.s004.tif]

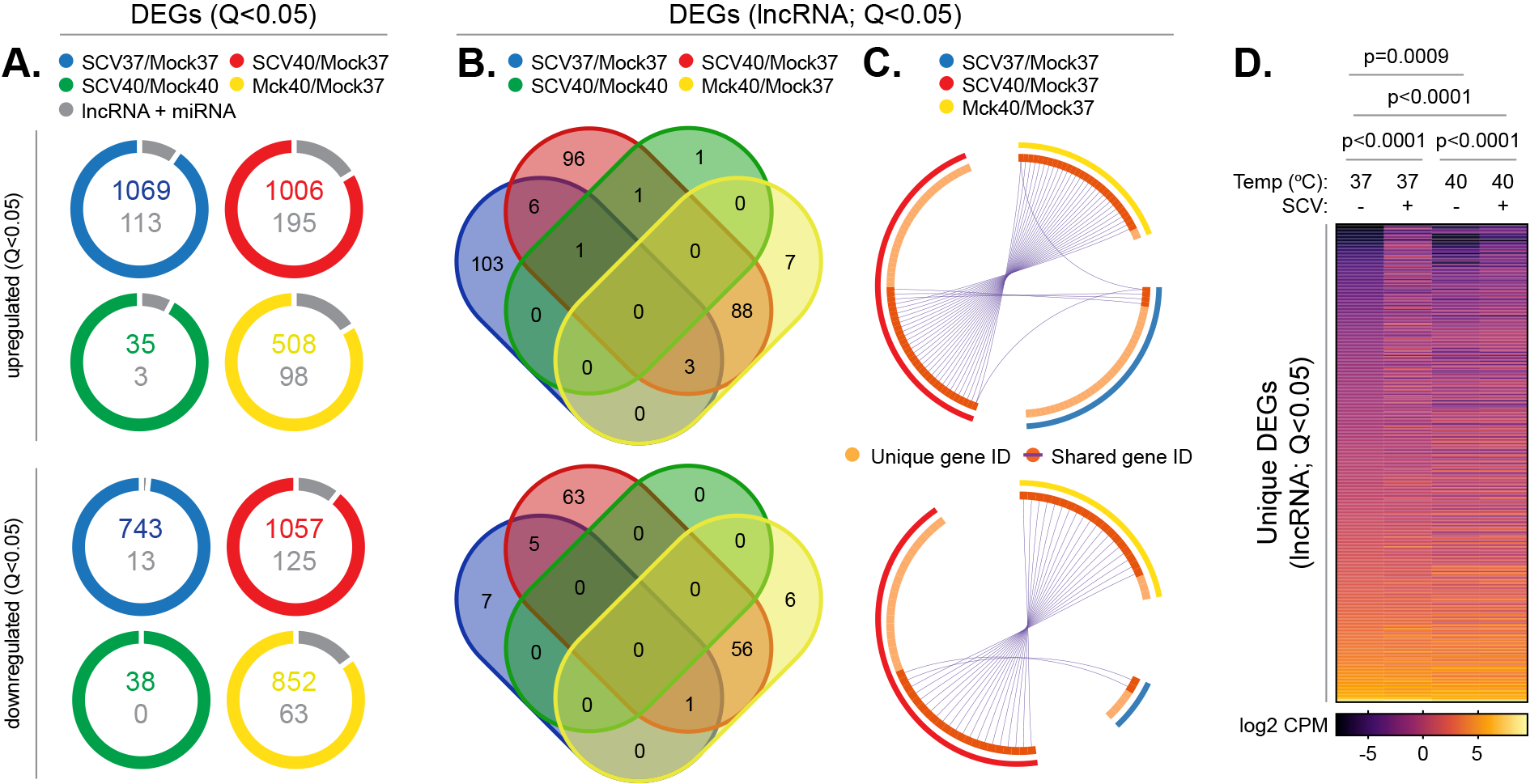

Supplement: S5 Fig — Ciliated respiratory cultures differentiated from primary HBEp cells isolated from 3 independent donors (donor 1, male Caucasian aged 63 years; donor 2, Hispanic male aged 62 years; donor 3, Caucasian female aged 16 years; all nonsmokers) were incubated at 37 or 40°C for 24 h prior to mock (media only) or SARS-CoV-2 (SCV2; MOI 0.05, 104 PFU/Tissue) infection. Tissues were incubated at their respective temperatures for 72 h prior to RNA extraction and RNA-Seq. High-confidence (Q < 0.05) DEG transcripts were identified (up-regulated [top panels] or down-regulated [bottom panels]) for each paired condition analyzed; blue circles/ellipses, SARS-CoV-2 37°C/Mock 37°C (SCV37/Mock37); green circles/ellipses, SARS-CoV-2 40°C/Mock 40°C (SCV40/Mock40); red circles/ellipses, SARS-CoV-2 40°C/Mock 37°C (SCV40/Mock37); yellow circles/ellipses, Mock 40°C/Mock 37°C (Mock40/Mock37). (A) Proportion of lncRNA (gray numbers and lines) DEGs identified per condition analyzed (colored numbers and lines). (B) Venn diagram showing the number of shared lncRNA identified between each paired condition analyzed. (C) Circos plot showing the proportion of unique (light orange inner circle) or shared (dark orange inner circle + purple lines) lncRNA between each paired condition analyzed. (D) Expression values (log2 CPM) of lncRNA identified per sample condition analyzed; p-values shown, paired two-tailed t test. (A to D) RNA-Seq data derived from RNA isolated from 3 donors (donors 1 to 3) per sample condition from 3 independent biological experiments per donor. Raw values presented in S7 and S9 Data. CPM, counts per million; DEG, differentially expressed gene; HBEp, human bronchiolar epithelial; lncRNA, long noncoding RNA; miRNA, microRNA; PFU, plaque-forming unit; RNA-Seq, RNA sequencing; SARS-CoV-2, Severe Acute Respiratory Syndrome Coronavirus 2. (TIF) [file pbio.3001065.s005.tif]

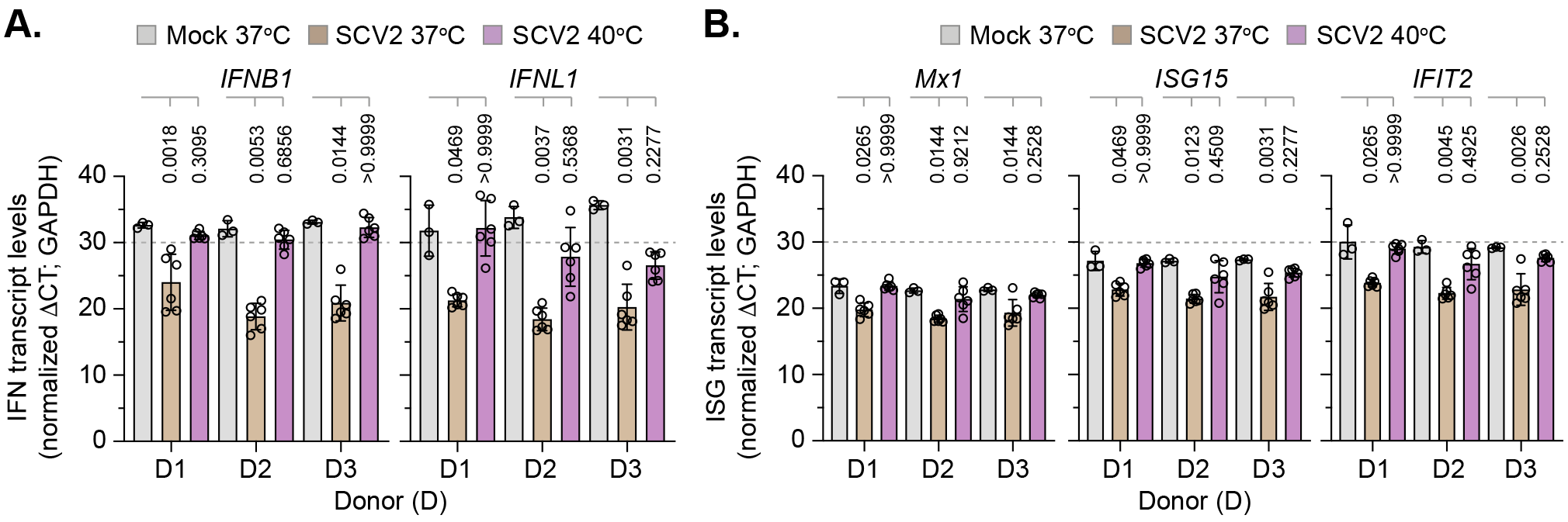

Supplement: S6 Fig — Ciliated respiratory cultures differentiated from primary HBEp cells isolated from 3 independent donors (donor 1 [D1], male Caucasian aged 63 years; donor 2 [D2], Hispanic male aged 62 years; donor 3 [D3], Caucasian female aged 16 years) were incubated at 37 or 40°C for 24 h prior to mock (media only) or SARS-CoV-2 (SCV2; MOI 0.05, 104 PFU/Tissue) infection. Tissues were incubated at their respective temperatures for 72 h prior to RNA extraction and RT-qPCR. (A/B) Quantitation of IFN (IFNB1 and IFNL1) or ISG (Mx1, ISG15, and IFIT2) transcript levels within mock of SARS-CoV-2–infected respiratory cultures. N ≥ 3 tissues per sample condition derived from a minimum of 3 independent biological experiments. Means and SD shown; all data points (normalized [GAPDH] ΔCT values) shown; p-values shown, one-way ANOVA Kruskal–Wallis test. Dotted line, threshold of linear assay detection. ΔCT values presented to aid comparison between donor samples relative to that of averaged ΔΔCT values presented in Fig 6E and 6F per experimental condition. Raw values presented in S9 Data. HBEp, human bronchiolar epithelial; IFN, interferon; ISG, IFN-stimulated gene; PFU, plaque-forming unit; RT-qPCR, reverse transcription quantitative PCR; SARS-CoV-2, Severe Acute Respiratory Syndrome Coronavirus 2. (TIF) [file pbio.3001065.s006.tif]

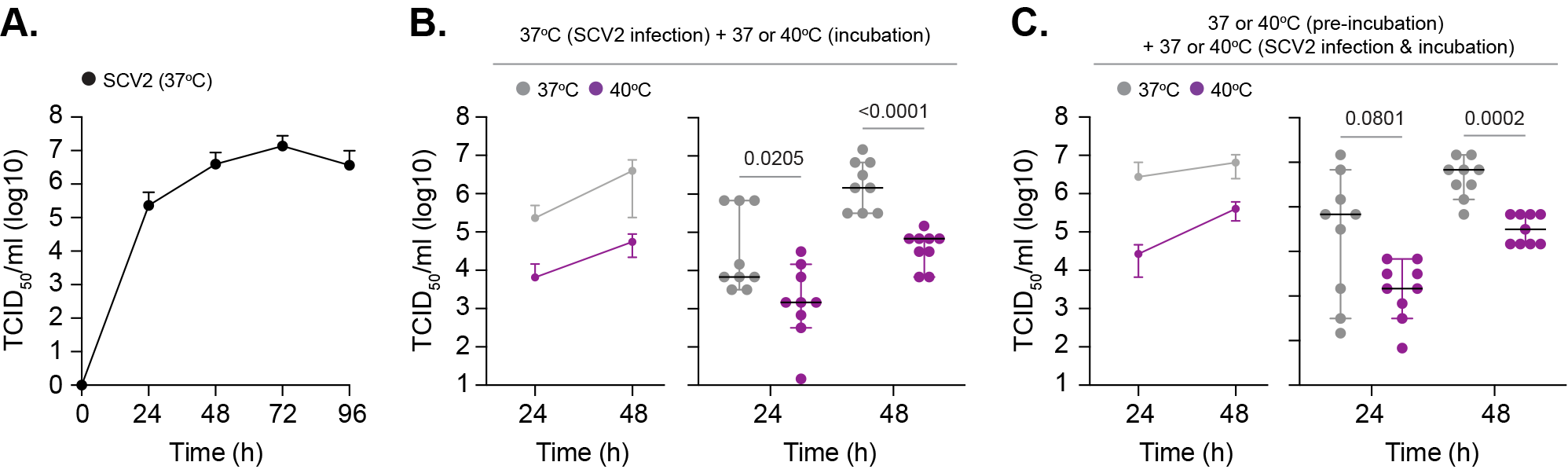

Supplement: S7 Fig — Vero E6 cells were infected with SARS-CoV-2 (SCV2; MOI 0.05 PFU/cell) at 37°C prior to temperature elevation and incubation at 37 or 40°C (A/B) or preincubated at 37 or 40°C for 24 h prior to infection and continued incubation at their respective temperatures (C). (A) TCID50 titers of supernatants derived from SARS-CoV-2–infected Vero E6 cells incubated at 37°C over time (h). Means and SD shown. (B/C) TCID50 SARS-CoV-2 titers at 24 and 48 h postinfection. Left-hand panel; means and SD. Right-hand panel; black line, median; whisker, 95% confidence interval; all data points shown; p-values shown, Mann–Whitney U test. (A-C) N = 3 independent biological experiments performed in triplicate. Raw values presented in S9 Data. PFU, plaque-forming unit; SARS-CoV-2, Severe Acute Respiratory Syndrome Coronavirus 2. (TIF) [file pbio.3001065.s007.tif]
