# Supplementary material for: Elevated temperature inhibits SARS-CoV-2 replication in respiratory epithelium independently of IFN-mediated innate immune defenses
Source: PLoS Biol. 2021 Dec 21;19(12):e3001065. doi: 10.1371/journal.pbio.3001065 (PMC8765667; doi:10.1371/journal.pbio.3001065)
Supplement: Attachment — Submitted filename: PLOS Biology response letter.docx [file pbio.3001065.s017.docx]

Dear PLOS Biology

As requested, please find below a point-by-point response addressing the reviewers’ comments and the revisions we have made to our manuscript "Elevated temperature inhibits SARS-CoV-2 replication in respiratory epithelium independently of the induction of IFN-mediated innate immune defences" (PBIOLOGY-D-20-03443R1). Line numbers below in our response are those associated with the track highlighted document. We would like to thank the reviewers for their constructive input. By addressing their concerns, we believe our manuscript to be significantly strengthened. We expect our study to have a broad readership appeal, as to our knowledge we present the first documented evidence that febrile temperatures associated with COVID-19 directly contribute to the cellular restriction of SARS-CoV-2 (SCV2) through a mechanism that influences viral transcription independently of the IFN response. Thus, we identify a previously unknown host response to coronavirus infection that contributes to an inhibition in SCV2 propagation that warrants detailed study from multiple labs to determine its relevance to COVID-19 disease progression.

We look forward to hearing your response to our pre-submission enquiry.

Sincerely

Chris Boutell, Pablo Murcia, and Sheila Graham.

Reviewer #1: The manuscript by Vanessa Herder, Kieran Dee, and colleagues demonstrates that SARS-CoV-2 replication is constrained at ≥39°C using different cell culture models. The results complement the increasing body of knowledge on SARS-CoV-2 replication in the human respiratory epithelium that likely can be extended to other pathogens and is therefore of potential interest to a broad audience. However, certain claims made by the authors are not completely supported by the data at hand and need to be further substantiated by additional experimental data, which I would like to clarify below.

Major:

C1) One of the most outstanding issues with the manuscript is that throughout the manuscript a large body of the data is based on invalid experimental design. For instance, the differential gene expression analysis is questionable as the RNA-seq analysis has unfortunately been performed on three technical replicates per condition (Lines 767 - 768, 783 - 784, 817 - 818, 836 - 837, 878 - 879, 891 - 892, 908 - 909, and 927 - 928), instead of three or more independent biological replicates per condition. Thus, the biological number of independent experiments equals one, and therefore, the data does not represent the biological variance, but rather the variance within a single sample. This should be resolved by complementing the existing data with additional biological replicates.

R1) Unlike traditional two-dimension (2D) cell culture systems, the differentiation of donor-isolated respiratory cells into three-dimensional (3D) respiratory epithelia is subject to inherent variance. Each primary population of donor-derived cells contains a mixture of progenitor, epithelial, fibroblast, and goblet cells, which vary in their respective proportion from source, population outgrowth upon expansion and transwell seeding, and differentiation state upon airlift. As there is no experimental way to normalise for this heterogeneity, each tissue is a unique entity of its itself. For these reasons, we differentiated respiratory epithelium from a single batch of donor cells for our RNA-Seq analysis to minimise variance upon tissue differentiation so that our analysis would only identify transcriptomic changes related to heat and/or SARS-CoV-2 (SCV2) infection. However, we do agree that RNA transcription levels could exhibit inter-individual variation. To address this reviewer’s concern (reproducibility), we have conducted RT-qPCR on independent batches of tissues derived from multiple donors to corroborate our major conclusions (now presented in Fig. 6E, F). These data validate our RNA-Seq analysis and show SCV2 infection at elevated temperature does not lead to the induction of IFN (*IFNB1*/*IFNL1*) or ISG (*Mx1*, *ISG15*, or *IFIT2*) expression. Thus, we show conclusive evidence that the temperature-dependent restriction of SCV2 occurs independently of IFN in a donor-independent manner. We have modified the manuscript throughout to reflect the addition of these data.

C2) In addition to the RNA-seq data, the statistical analysis on the viral replication kinetics has been done using the technical replicates (e.g N=300, N=5, N=11 or N=9) rather than biological replicates (N=3)
(line 743, 792 - 793, 797, 800 - 801, 847 - 848). Please clarify this and revise the statistical analysis throughout the manuscript accordingly.

R2) As outlined above, each tissue represents a unique biological entity. As such, and in line with PLOS Biology guidelines, we present every data point derived over three independent biological experiments; as opposed to the means of replicates that assume each tissue to be equivalent in its cellular composition, differentiation state, and response to SCV2 infection. As differentiation invariable leads to unequal populations, due to a loss of transwells over a four-week differentiation period, we have performed non-parametric statistical tests throughout the study to avoid biasing the data through the removal of unpaired data points. We believe this approach to be the most transparent and accurate way of presenting the data. The ‘N=300’ refered to by the reviewer comes from the total number of epithelial thickness measurements conducted previously in Figure 3. This data has now been removed and replaced by transepithelial electrical resistance (TEER) measurements that directly measure epithelial membrane integrity as a proxy of epithelium health (see below). We now define ‘N’ to be ‘the number of tissues sampled per experimental condition derived from a minimum of three independent biological replicates (unless otherwise stated)’ in the statistical analysis section of the material and methods and figure legends throughout.

C3) Despite the aforementioned discrepancies, this reviewer acknowledges that the authors elegantly demonstrate on different levels that an elevated temperature (39°C/40°C) alters the in vitro respiratory epithelium and constrains SARS-CoV-2 replication in different cell culture models. However, the reviewer is not convinced by the claim that restriction of SARS-CoV-2 is independent of the induction of type-I and type-III IFN-mediated antiviral immune defenses. This as the authors do not directly and conclusively demonstrate that SARS-CoV-2 (i) enters the respiratory epithelium and (ii) that there is robust productive viral replication in (iii) the absence of an innate immune response. This is for instance exemplified by the results that there are no antigen-positive cells for SARS-CoV-2 detected at 40°C (Figure 8E). Moreover, it cannot be excluded that the low concentration of viral RNA detected at 40°C reflects residual virus from the inoculum (which is missing from the
figures), as the apical surface is only washed once after a 2 hour inoculation (Line 375 - 378). In line with this, the viral replication kinetics (TCID50) at 72 hours are all likely in proximity to or below the limit of detection of the assay and might reflect residual virus from the inoculum.
R3-i) We now present RT-qPCR data demonstrating that RNA isolated from infected tissues at 40°C from multiple donors to contain SCV2 RNA at levels greater than that of the 0 h time point (an apical wash collected immediately after removal of inoculum). Moreover, we show infectious viral titres at 72 h to also be higher than that of the 0 h time point. Together, these data demonstrate the respiratory epithelium to be permissive to infection at elevated temperature, but refractory to SCV2 RNA replication relative to infection at 37°C.

R3-ii) We now show the block in SCV2 replication to occur at a transcriptional level (see below) that correlates with reduced levels of intraepithelial vRNA replication (as determined by both RT-qPCR and in situ hybridisation). These data are consistent with our RNA-seq analysis, which shows significantly reduced levels of viral gene expression within tissues infected at elevated temperature. This is consistent with our viral antigen staining, which shows reduced numbers of viral antigen foci at 40°C.

R3-iii) We now present RT-qPCR data on RNA extracted from infected tissues from three independent donors to corroborate our RNA-Seq analysis. These data significantly strengthen our claim that the temperature-dependent restriction of SCV2 occurs independently of, and prior to, the induction of IFN-mediated antiviral immune response. Moreover, inhibition of IFN signalling by Ruxolitinib, an inhibitor of Janus-associated kinase (JAK), does not significantly influence SCV2 apical titres by 72 h post-infection in our model system (response Figure 1 below). Thus, the restriction observed at 40°C in the absence of detectible levels of IFN induction occurs independently of IFN-mediated immune defences.

Therefore, in order to support the major claim that "the restriction of SARS-CoV-2 is independent of the induction of type-I and type-III IFN-mediated antiviral immune defenses", the reviewer would like to ask the authors to provide the following experimental data: 
1.      Validation that SARS-CoV-2 enters and productively infects the cultures, by using a PCR specifically targeting the leader-body junction of a sub-genomic mRNA of SARS-CoV-2.  We now present RT-qPCR data using two independent primer probe sets that show intracellular levels of vRNA to be higher than that of the T=0 h apical wash. Importantly, these tissues have been sequentially washed over the time course of infection. Thus, we demonstrate tissues incubated at 40°C to remain permissive to viral entry with limited levels of SCV2 replication in a donor independent manner.

2.      Monitoring of the viral kinetics and host response in an experiment whereby during the course of the experiment the temperature is being elevated from the core body temperature (37°C) to 40°C in the absence or presence of the Janus-associated kinase (JAK) inhibitor Ruxolitinib. We have performed a pilot experiment (Response Figure 1) examining the influence of Ruxolitinib on SCV2 replication at 37°C under the same infection conditions used throughout the study. Inhibition of JAK had no significant effect on SCV2 apical titres by 72 h post-infection. Thus, the full impact of IFN-mediate antiviral immunity has yet to take effect at this stage of infection; time points at which we show SCV2 temperature restriction. These data are consistent with findings that have shown SCV2 to induce a relative weak immune response (Hadjadj et al 2020; DOI, 10.1126/science.abc6027). Importantly, we now show that SCV2 infection at 37°C for 24 h prior to thermal upshift to 40°C also leads to significant levels of SCV2 restriction. Thus, we demonstrate that the thermal restriction of SCV2 infection occurs independently of a potential block to viral entry and IFN-mediated antiviral immune defences. We thank the reviewer for this suggestion. As we present data examining thermal upshift post-infection and expression of IFN and ISGs from multiple donors at both 37 and 40°C (described above in R1), the Ruxolitinib experiment below is redundant and presented for reference only.

|  | **Response Figure 1: Inhibition of the IFN response by Ruxolitinib does not lead to enhanced viral titres by 72 h post-infection.**  Ciliated respiratory cultures differentiated from HBEp cells isolated from donor 1 (male Caucasian aged 63) were treated with DMSO or Ruxolitinib (Ruxo; 5μM) for 1 hour prior to SCV2 infection (MOI 0.05, 10^4^ PFU/Tissue) and incubation in the presence or absence of inhibitor at 37°C. Apical washes were collected over time (as indicated) and viral titres calculated by RT-qPCR. Means and SD shown; p-values shown, Mann-Whitney *U*-test. N=9 tissues per sample condition derived from three independent biological experiments. |
| --- | --- |

Minor:
Please include the viral inoculum in all of the replication kinetic figures throughout the manuscript.

We have now included a T= 0h (titrated apical wash collected immediately after removal of inoculum) on all replication kinetic data presented. Unlike the inoculum titre, the T= 0h titre directly correlates to surface bound residual virus. The inclusion of these data points greatly improves the independent assessment of the replication kinetics of SCV2 upon temperature elevation relative to input. We thank the reviewer for this suggestion, as it significantly improves visual data interpretation aiding to the strength of our conclusions.

Figure1B and E, why is there a drop in viral titer at 120 hpi?

The drop we observe in infectious viral titre is consistent with a previous study that has shown SCV2 release from respiratory epithelia to occur in waves over prolonged periods of incubation (Hao et al., 2020; 10.1128/mBio.02852-20). We have added this to the description of these data (lines 385 to 387).

Line 353 "VeroE6" should be written as "Vero E6"

We have made this change throughout the manuscript.

Figure 5B please include a color legend for the Chord diagram, in either the figure or figure legend.
We have included a colour legend as requested

Reviewer #2: The manuscript by Herder et al. studies the effect of elevated temperature on SARS-CoV-2 replication. Elevated temperature mimics the fever, which is one of the most frequent symptoms of SARS-CoV-2 induced disease (COVID-19). 

Using an experimental model that consists in differentiated primary human bronchial 3D epithelial (HBE) cells grown under air-liquid interface (ALI), the replication of SARS-CoV-2 and the cellular gene expression profile were analyzed at the physiological temperature of 37ºC and at the hyperthermic temperatures of 39ºC and 40ºC. RNAseq results showed that temperature elevation induced a heat stress response in the respiratory tissue model. It also restricted the replication of SARS-CoV-2, as confirmed by RNAseq and titration of infectious virus. The significant reduction of virus replication at 40ºC failed to induce the production of interferon. 

The experimental model and the design of the experiments are appropriate. However, the novelty of the results is limited. As mentioned in the manuscript, the analysis of the cellular pathways differentially expressed in response to SARS-CoV-2 infection (Fig. 2) has been previously reported (references 16, 18-21, 30-32). It has also been extensively described that the temperature elevation induces in tissues a heat stress response (Fig. 3).  
We agree with the reviewer that Fig.2 (the induction of IFN signalling pathways) is somewhat redundant and have moved this into supplemental information (Fig. S2). However, while the heat-stress response has been extensively studied in 2D cell culture, we can find no reference to its study in 3D respiratory epithelial tissue culture. It therefore remains important to formally demonstrate that this host response is activated in our model system independently of the production of DAMPs (damage associated molecular patterns) that could lead to the induction of an IFN response which may influence the interpretation of viral infection studies. We have corrected the manuscript to make this rationale clearer (lines 390 to 392).

The most novel aspect of the manuscript is the description of reduced viral RNA levels at elevated temperatures, which leads to a reduction in viral titers (around 2-log units lower at 40ºC than at 37ºC). This effect has been previously described in influenza virus as a result of decreased thermal stability of the RNA-dependent RNA polymerase. However, in coronaviruses, the impact of temperature elevation has not been analyzed in depth. The mouse hepatitis coronavirus replicates to high titers both at 37º and 40°C, whereas a temperature-sensitive mutant with mutations in the nsp3 showed replication defects at 40ºC. The relevant observation that the replication of a clinical isolate of SARS-CoV-2 is sensitive to temperature would require further research to characterize the mechanisms responsible for this sensitivity.

We agree with the reviewer that additional research out with the scope of this study is required to define the precise molecular mechanism(s) of restriction. However, we now present evidence to show that this block is likely to occur at a transcriptional level (see below). We therefore present the first evidence to show febrile temperatures associated with COVID-19 lead to a cellular restriction in SCV2 replication through a mechanism that influences viral transcription. This mechanism occurs independently of IFN-mediated antiviral immune defences known to restrict SCV2 replication. Thus, we identify a novel host response to coronavirus infection that contributes to an inhibition in SCV2 propagation that warrants detailed study from multiple labs to determine its relevance to COVID-19 disease progression.

Specific points

1.      Primary human bronchial epithelial cells from one single human donor were used for the experiments. Because of the high inter-donor variability, samples from several donors should have been used in order to achieve more representative and conclusive results.

R1). We have now repeated our analysis in respiratory tissues derived from two additional donors. We now show the temperature-dependent restriction of SCV2 to occur in respiratory epithelia derived from three independent donors in an IFN-independent manner. Notably, we do identify donor dependent phenotypes in the thermal restriction of SCV2. These data are now presented in Fig. 4. We have modified the manuscript throughout to take into account these new data.

2.      The impact of the higher temperatures in the viability of the cells should be analyzed to differentiate whether the restriction of viral replication is a direct effect on the activity of viral proteins or an indirect effect mediated by a reduced cell viability.

R2). We have performed transepithelial electrical resistance (TEER) measurements to demonstrate respiratory epithelia incubated at 40^o^C retains epithelial integrity at 72h post-treatment as a proxy for epithelium health (Fig. 2B). Additionally, RNA-Seq data demonstrates that cellular transcription of a reference set of genes that are expressed across a wide range of tissues and cell-types remains unperturbed at elevated temperature. No significant DEG enrichment was observed in necrotic or apoptotic pathways to indicate reduced levels of viral replication occurred as a consequence of tissue viability. We have performed Periscope analysis (Parker et al., 2021; DOI, 10.1101/gr.268110.120) on our RNA-Seq data. This analysis has identified viral sub-genomic RNA (sgRNA) expression to be significantly reduced in tissues infected at 40^o^C (Fig. 7E). As the expression of viral sgRNAs requires the onset of viral transcription, we have identified the thermal restriction of SCV2 to occur at the transcriptional level. We have modified the results section (lines 821 to 825) and discuss these data in the context of the viral RdRp activity and binding affinity in the discussion of our manuscript.

3.      Are there any mutations in consensus sequence of the virus after the high-temperature incubation period?

R3) We have sequenced our input virus and extracellular virus collected from apical washes collected at both 37 and 40 ^o^C. The analysis of which confirms the block to viral replication to occur independently of viral mutation (Table 1). We have modified the manuscript throughout to discuss this analysis.

4.      The mechanisms involved in the reduced viral growth should be studied by analyzing whether they are acting at the level of entry, viral RNA synthesis, protein synthesis, morphogenesis…
R4) As stated above (R2), we have performed Periscope analysis on our RNA-Seq data to examine the expression of viral sgRNAs which has identified the temperature-dependent restriction in SCV2 infection to occur at a transcriptional level. These data are consistent with our RNA-scope and IF analysis, which fail to detect SCV2 vRNA replication and viral antigen expression within tissues infected at 40^o^C. As stated in our discussion, due to the number of pathways observed to be induced with respiratory epithelia incubated at elevated temperature we cannot discount the possibility that multiple pathways contribute to the observed temperature-dependent restriction in SCV2 replication. However, our analysis clearly shows there to be a prominent block in viral transcription.

Minor points

5.      Fig. 1B and 1C graphs showing all data points or means and SD, respectively, are redundant and should be unified. The same is true for Fig. 4A-B and Fig. for 4C-D.

R5) We have removed the duplication of figures throughout the manuscript.

6.      Fig. 1. The amount of virus inoculum in infections should be indicated as a multiplicity of infection (MOI=PFU per cell) instead of PFU per transwell.

R6) As it remains unknown how MOI (PFU per cell) directly relates to SCV2 infection within a 3D epithelium we now present both. We did not wish to give the impression or presumption that each cell will take up an equivalent amount of virus from the inoculum, which is only delivered to the apical surface. Moreover, we observed specific areas of the epithelium to initiate SCV2 replication which subsequently propagates in an intraepithelial fashion (Figure 1, RNA-scope). We have modified the manuscript to describe these observations more accurately.
