# Supplementary material for: Elevated temperature inhibits SARS-CoV-2 replication in respiratory epithelium independently of IFN-mediated innate immune defenses
Source: PLoS Biol. 2021 Dec 21;19(12):e3001065. doi: 10.1371/journal.pbio.3001065 (PMC8765667; doi:10.1371/journal.pbio.3001065)
Supplement: Attachment — Submitted filename: Response letter_R4.docx [file pbio.3001065.s018.docx]

Dear PLOS Biology

As requested, we have uploaded our revised manuscript and point-by-point response addressing the reviewers’ comments to our previously submitted manuscript "Elevated temperature inhibits SARS-CoV-2 replication in respiratory epithelium independently of the induction of IFN-mediated innate immune defences" (PBIOLOGY-D-20-03443R3). Line numbers in our response are those associated with the track accepted document. We would like to thank the reviewers for their constructive input, as the additional RNA-seq data significantly strengthens our study design and major conclusions. We expect our study to have a broad readership appeal, as we present the first documented evidence that febrile temperatures associated with COVID-19 directly contribute to the cellular restriction of SARS-CoV-2 during infection of respiratory epithelial tissue through a mechanism that inhibits viral transcription independently of the IFN response. Thus, we identify a previously unknown host response to coronavirus infection that contributes to an inhibition in SARS-CoV2 propagation that we expect to have a wide readership appeal.

We look forward to hearing your response to our resubmission.

Sincerely

Chris Boutell, Pablo Murcia, and Sheila Graham.

17.11.2021

Response to reviewers comments:

Reviewer #1: The reviewer acknowledges and values the extensive revision that is presented by Vanessa Herder, Kieran Dee, and colleagues. This has increased the overall study, and thereby reviewer is still convinced that the concept is of interest to a broad scientific audience. However, the reviewer regrets that the current way of data presentation and statistical testing are inappropriate to validate whether the additional experimental data confirms the earlier observations made with the RNA-seq analysis, which is regrettably still a single independent biological donor. This crucial point requires specific attention from the authors and is described in more detail below. As requested by the reviewer we have now repeated the RNA-seq analysis from three independent donors (see response below).

Major:
**C1)** The reviewer would like to thank the authors for the detailed explanation on how the statistical analysis was conducted and hopes that the commercial multipotent basal airway cells to establish differentiated airway epithelial cell cultures are not contaminated with fibroblasts.

**R1)** We can assure the reviewer that this is not the case, as evidence by the representative H&E stained tissue sections in the manuscript (Figures 1 and 4)

**C2)** However, as indicated above, the reviewer unfortunately strongly disagrees on the appropriateness of the statistical testing and underlying scientific reasoning of the authors. This because measurement of individual wells from the same biological donor, differentiated in parallel from the same batch of cells and used at the same instance, need to be considered as repeated measurements of the noise (e.g., cellular heterogeneity) associated with the plastics, culture reagents and handling procedures. Irrespective whether this is conventional or more advanced primary cell culture. In a more simplified analogy, one cannot consider a single 96-well cluster plate with Vero E6 cells obtained from a single
culture flask, that are all infected with SARS-CoV-2 at the same time as 96 individual biological replicates. Instead, this is a single independent biological replicate (N=1) with 96 technical replicates, which all have an inherent cellular heterogeneity (e.g., distinct receptor expression profiles, cell cycle, etc.). Hence, if primary airway cells of a single biological donor originating from a culture flask are divided over different transwell inserts, these individual inserts from the same batch and donor need to be considered as technical replicates, as they do not represent biological variation.
Therefore, the reviewer urges the authors to include more donors for the RNA-seq analysis (such as from the samples included in the revision), and stresses that the authors update their data and statistical analysis and presentation throughout the manuscript. This to reflect the actual biological variation from the 3 different donors used in the manuscript, rather than stratifying the data by donor and performing statistical analysis on technical replicates for each individual donor (e.g., N=9 in Fig3, 4A-C, and Fig 6E-F). This, as the latter needs to be considered as "pseudo-replication", the inflation of the actual number of samples, which does not reflect the actual biological variation the authors would like to interpret their data from, nor want to convey to the scientific audience.
**R2)** As requested by the reviewer we have repeated the RNA-seq analysis for all three independent donors. Principle component analysis (PCA) analysis of mean CPM values derived from three independent experiments per sample condition per donor demonstrates donor specific variation in their respective host responses to SCV2 infection (Figure S1). We have revised our transcriptome analysis throughout the manuscript to reflect the incorporation of these new data, which significantly strengthen the overall study design. As these data have been normalized, we have performed parametric tests throughout the study regarding the analysis of transcriptome data and revised RT-qPCR data (which is also normalized; Figure 6E/F). We are happy to report that our major findings and conclusions hold true, although we now detect a weak immune signature during SARS-CoV-2 infection at 40^o^C due to differential expression of 9 ISGs relative to mock treatment (Figure S3). We now present our Ruxolitinib analysis (Figure 6G) to demonstrate that even the highest levels of immune stimulation observed at 37^o^C have negligible impact on SARS-CoV-2 replication at 72 hpi in our model system. These data add supporting evidence to our conclusions that the restriction of SARS-CoV-2 observed at elevated temperature between 24 and 72 hpi occurs independently of IFN-mediated host immune defences. We have revised the manuscript throughout to take into account the inclusion of these new data. It is currently the policy of PLOS biology to show all data points. As stated in our previous response letter, a four-week differentiation period invariably leads to unequal populations of data points due to the loss of respiratory cultures grown in the absence of antibiotics. Thus, we have performed non-parametric statistical tests throughout the study on all viral replication data, which cannot be normalized, to avoid data manipulation through the removal of data points. If the journal deems it appropriate to do so, we are happy to combine our replicate experimental data as suggested by the reviewer. However, we believe the journal’s current policy of showing all data points to be the most transparent way of presenting the data derived from respiratory cultures, which show inherent variance in the levels of SARS-CoV-2 replication (≥ 10-fold variation per experimental condition at 72 hpi; Figure 4B) between infected tissues irrespective of the donor from which the cells were isolated from. Notably, due to the divergence observed between donor samples (irrespective of experimental condition, see PCA plot in Figure S1), non-parametric testing would still be the most appropriate statistical analysis for testing non-normalized virus replication data which show donor dependent profiles of viral replication at either 37 or 40^o^C (Figure 4B).

**C2)** The graphs in Fig 6E-D, are not intuitive to understand and need to be reanalysed using the more appropriate delta-delta Ct approach (<https://doi.org/10.1006/meth.2001.1262>), and should reflect the actual biological variation.
**R2)** We now present our RT-qPCR data as delta-delta Ct (Figure 6G). Parametric analysis of these normalized data supports our previous conclusions. However, we still believe it is informative to reader to be able to compare the variance in IFN and ISG induction between infected tissues differentiated from individual donors or between donors. Thus, we have moved our previous analysis into Figure S6 for reader comparison.

**C3)** The statement in line 147 - 148 is incorrect, as it has previously been shown that temperature restricts SARS-CoV-2 replication, as the authors themselves also highlight in the introduction Line 68 - 71. This should be correct to for instance: "To our knowledge, these data for the first time demonstrate that febrile temperatures restrict SARS-CoV-2 replication…."
R3) We thank the reviewer for bringing this to our attention. We have now revised this statement to read; ‘These data identify febrile temperature ranges associated with low (39°C) to moderate (40°C) grade fever to restrict SARS-CoV-2 propagation in respiratory epithelia’ [lines 145 to 147]. We have also corrected a similar error in the discussion, which now reads; ‘While SARS-CoV-2 has been reported to replicate to higher viral titres at temperatures associated with the upper respiratory airway (33°C) relative to core body temperature (37°C) [16], the replication of coronaviruses at febrile body temperatures has remained poorly characterized’ [lines 325 to 328].

Minor:
- It does not make sense to abbreviate the well-established abbreviation SARS-CoV-2.

- We have replaced the SCV2 abbreviation throughout the main body of the text in the manuscript. However, we have retained this abbreviation for the figures and tables for spacing issues. The SCV2 abbreviation is defined in the figure/table legend in these instances.

- Line 397; 1.1x10E5 cells/well should this not be ten-fold lower?

- We thank the reviewer for bring this error to our attention and have modified the text accordingly. Cell stocks were made up to 1.1x10e5 cells/ml. 96 well plates were seeded with 100 μl of cell suspension (1.1x10e4 cells/well).

**Reviewer #2:** Most of the points raised by this reviewer have been satisfactorily addressed in the revised version of the manuscript by Herder et al.

**C1)** However, there is one new point that should be clarified. It is claimed in the Abstract that temperatures >39ºC restrict viral growth by limiting viral transcription. This statement is supported by the RNA-seq results shown in Fig. 7D and 7E.

**R1)** We thank the review for bring this statement to our attention. We have revised the text to now read; ‘Respiratory tissue incubated at 40°C remained permissive to SARS-CoV-2 entry but refractory to viral transcription, leading to significantly reduced levels of viral RNA replication and apical shedding of infectious virus’ [lines 34 to 36].

**C2)** RNA synthesis in coronaviruses include two differentiated processes, replication, which copies the whole genomic RNA (gRNA), and transcription, which synthesizes subgenomic mRNAs (sgmRNAs) of 3' end genes by a discontinuous mechanism. Regulation of sgmRNA levels does primarily depends on the levels of genomic RNA acting as templates for transcription, in addition to specific transcription-regulating factors.
Then, for sgmRNA quantification, the read counts of sgmRNAs should be normalized to the levels of gRNA in order to analyze whether or not lower levels of sgmRNAs are just a direct consequence of reduced gRNA levels. That being the case, a specific regulatory effect of temperature on transcription could not be confirmed.

Therefore, sgmRNAs (Fig. 7E) should be normalized to gRNA levels, which are determined from the read counts of Orf1ab, only produced by replication and not by transcription. In fact, including Orf1a in Fig. 7E as a sgmRNA is not correct, since Orf1a is not synthesized by the transcription mechanism.

In summary, to conclude an effect of tissue temperature specifically on viral transcription, a reduction on sgmRNA levels, further than that derived from the reduction in template gRNA levels should be confirmed.

**R2)** We agree with the point the reviewer raises. We now present normalised sgRNA MR counts as requested (Figure 7E). Analysis of these normalised data still identify sgRNA MR counts to be significantly reduced during SARS-CoV-2 infection of respiratory cultures at 40°C. Thus, our conclusion still stands.
